# The Integration of Multiplex PCR Panel in the Management of Acute Bacterial Meningitis: A Mixed-Methods Study

**DOI:** 10.3390/microorganisms14061279

**Published:** 2026-06-05

**Authors:** Olimpia-Catrinel Militaru, Laura-Elena Marin, Raluca-Mihaela Matoru, Daniela Tălăpan, Cristian-Mihail Niculae, Adriana Hristea

**Affiliations:** 1 Faculty of Medicine, Carol Davila University of Medicine and Pharmacy, 020021 Bucharest, Romania; olimpia-catrinel.ciuca@drd.umfcd.ro (O.-C.M.); daniela.talapan@umfcd.ro (D.T.); cristian.niculae@drd.umfcd.ro (C.-M.N.); adriana.hristea@umfcd.ro (A.H.); 2 “Prof. Dr. Matei Bals” National Institute for Infectious Diseases, 021105 Bucharest, Romania; raluca-mihaela.matoru@rez.umfcd.ro; 3 Colentina Clinical Hospital, 020125 Bucharest, Romania

**Keywords:** bacterial meningitis, multiplex PCR, meningitis BioFire, antimicrobial stewardship, qualitative research, mixed-methods

## Abstract

Meningitis multiplex PCR (MMP) panels are increasingly used in acute bacterial meningitis (ABM), but their clinical integration remains incompletely characterized. We evaluated MMP panel implementation using a mixed-methods approach. We included 55 adults with ABM of confirmed etiology: Group 1 (MMP plus conventional microbiology, *n* = 25) and Group 2 (conventional methods only, *n* = 30). The qualitative component comprised semi-structured interviews with infectious disease specialists, analyzed using thematic analysis. Compared with Group 2, Group 1 had a significantly shorter time from lumbar puncture to diagnosis [1.9 (IQR 1.7–2.6) vs. 27.3 (18–47.2) h] and more frequent targeted therapy [19 (76%) vs. 13 (43.3%)]. However, MMP panel use was not associated with antibiotic de-escalation [11 (44%) vs. 12 (40%)], median length of hospitalization (22 days in both groups), median duration of therapy [14 (10–21) vs. 17 (11–22) days], ICU admission [11 (44%) vs. 13 (43.3%)], or mortality [2 (8%) vs. 6 (20%)]. Interviews (*n* = 20) identified four themes: rapid etiological clarification, perceived limitations, antimicrobial optimization and clinical integration. MMP may facilitate rapid diagnosis, but its impact on outcomes and clinical integration remains limited. Faster availability of etiological information does not necessarily improve antimicrobial decision-making in the absence of antimicrobial stewardship programs.

## 1. Introduction

Acute bacterial meningitis (ABM) is a medical emergency in which rapid identification of the causative pathogen is essential for targeted therapy and improved clinical outcomes. Traditional microbiological cerebrospinal fluid (CSF) analysis remains fundamental, but it is time-consuming and may yield false-negative results, particularly in patients previously exposed to antibiotics. Molecular diagnostic techniques like multiplex PCR assay (BioFire FilmArray Meningitis/Encephalitis—BioFire Diagnostics, LLC, a bioMérieux company, Salt Lake City, UT, USA) enables rapid identification of the most common pathogens from CSF with high sensitivity (90–92%) and specificity (97–99%) [1]. However, the real-world interpretation and integration of these tools into clinical decision-making remain insufficiently characterized, and data on guideline adherence and variability in molecular testing implementation are scarce.

We performed a mixed-methods investigation. Through quantitative instruments, we aimed to assess the diagnostic performance of meningitis multiplex PCR (MMP) panels when compared to the classical diagnosis methods (CSF culture) in ABM, in terms of duration to the etiological diagnosis, clinical outcome (duration of hospitalization and antibiotic treatment, mortality, intensive care unit admission) and treatment optimization (antibiotic stewardship). In addition, using a qualitative approach, we aimed to gain an in-depth understanding regarding infectious disease physicians’ perspectives on the clinical value and limitations of MMP panels, when it comes to the process of diagnostic and therapeutic decision-making. By combining the quantitative and qualitative results, we sought to obtain meaningful insights into physicians’ process of integrating MMP panels into everyday clinical practice which are mandatory when it comes to enhancing the rational implementation of molecular testing in ABM.

## 2. Materials and Methods

The study was conducted using a convergent mixed-methods design, which is characterized by the fact that quantitative and qualitative data are collected and analyzed separately. In order to achieve a more comprehensive analysis, the final step of the process of data interpretation in this approach consists mixing the results from both methods [2].

### 2.1. Quantitative Approach

#### 2.1.1. Methodology and Population

The quantitative study was a retrospective analysis conducted in a tertiary referral center specialized in infectious diseases, The National Institute for Infectious Diseases “Prof. Dr. Matei Balș”, from Bucharest, Romania. Data were collected using electronic medical records of patients admitted between January 2019 and December 2023.

We included adult patients defined by ≥18 years old, hospitalized with a diagnosis of ABM, and further excluded the cases that did not have an identified etiological agent. All patients had a positive diagnosis based on the presence of standardized clinical criteria for meningitis (fever, headache, meningeal irritation signs, and/or signs of increased intracranial pressure) and abnormal CSF analysis on routine tests (cell count, cell differential, elevated protein levels, and a low serum-to-CSF glucose ratio) [3,4,5]. The etiology was established using traditional bacterial cultures for all patients, and, for a part of them, the meningitis/meningoencephalitis multiplex PCR assay (Meningitis/Encephalitis BioFire^®^ panel-BioFire Diagnostics, Salt Lake City, UT, USA). This molecular technique automatically extracts and processes the nucleic acids for common bacterial pathogens (*Streptococcus pneumoniae*, *Haemophilus influenzae*, *Neisseria meningitidis*, *Listeria monocytogenes*, *Streptococcus agalactiae*), viruses (enterovirus, herpes simplex virus type 1 and 2, varicella-zoster virus, cytomegalovirus, human herpes virus 6 and human parechovirus) and fungi (*Cryptococcus* spp.) [6].

**Figure 1:**
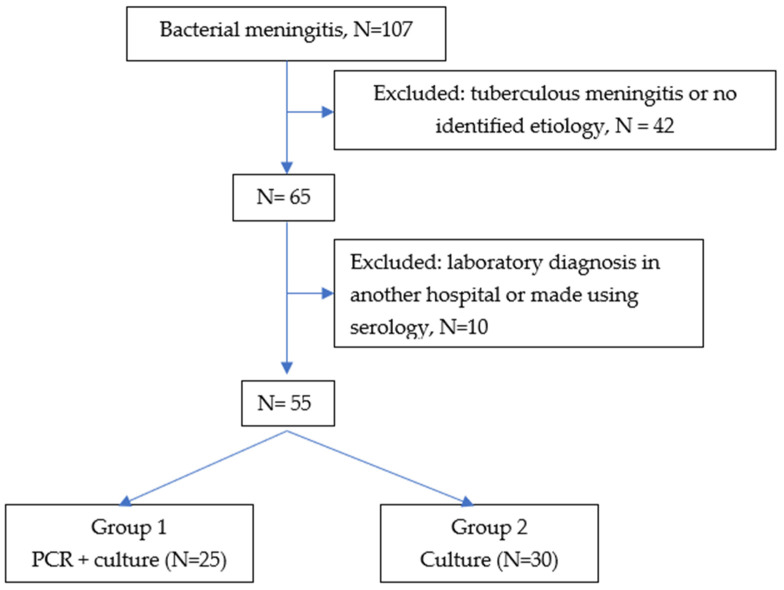
Flow diagram illustrating the study exclusion criteria.

Patients with a confirmed diagnosis of tuberculous meningitis were excluded from the study, as were patients whose etiological diagnoses were established at external institutions or based on serological testing, as described in Figure 1.

#### 2.1.2. Data Collection and Statistical Analysis

Eligible patients were classified into two groups, according to the use of the MMP panel: Group 1 with diagnosis made by both MMP panel and CSF bacterial culture and Group 2 with diagnosis made using the traditional CSF culture alone.

Group 1 comprised 25 patients; this relatively limited sample size was influenced by the constraints imposed during the Coronavirus Disease of 2019 (COVID-19) pandemic, during which our hospital exclusively managed patients infected with severe acute respiratory syndrome coronavirus 2 for 26 months, followed by a period in which the majority of admissions were also COVID-19 cases. Group 2 included 30 patients that were mostly diagnosed in 2019, when MMP panel testing was not routinely available in our hospital; therefore, the diagnosis was made using traditional CSF culture alone.

For each group, we recorded demographic data, time to etiological diagnosis (inferred from result timestamps), empiric and targeted antibiotic, duration of antibiotic treatment, treatment adequacy, length of hospitalization, intensive care unit admission, and mortality.

Treatment adequacy was established using current meningitis guidelines, the 2025 World Health Organization guidelines and the 2016 European Society of Clinical Microbiology and Infectious Diseases guidelines for empirical and targeted antibiotic treatment [7,8].

Adequate empiric antibiotic therapy was defined as any one of the following regimens: (1) vancomycin plus ceftriaxone (or another third- or fourth-generation cephalosporin) with ampicillin/amoxicillin, if risk factors for *Listeria* were present: age over 60 years old, ongoing pregnancy, immunosuppressive therapy, organ transplantation, presence of malignancy, advanced HIV infection, diabetes mellitus, advanced chronic kidney disease, liver cirrhosis, chronic alcoholism [7,8]; (2) vancomycin plus aztreonam and/or trimethoprim–sulfamethoxazole; (3) any other antibiotic regimen with clinician documentation of intended bacterial meningitis coverage. In the third category, we included two cases: in one case of *K. pneumoniae* meningitis, meropenem was considered an adequate regimen because blood cultures positive for *Klebsiella* spp. were already available at the time of treatment initiation. Additionally, in a case of nosocomial *Acinetobacter baumannii* meningitis, the combination of meropenem and polymyxin E was classified as adequate given the high prevalence of extensively drug-resistant *Acinetobacter* strains in Romania, which justified broad-spectrum coverage pending susceptibility results.

Adequate targeted therapy prior to antibiogram availability was defined as any tailoring of antibiotic therapy based on positive microbiology results (Table 1). Examples of optimization/de-escalation might include switching to an antibiotic regimen directed towards a detected bacterial pathogen according to the expected susceptibility profile of the identified pathogen, without unnecessary coverage for other bacteria [9].

Inadequate empiric or targeted antibiotic therapy was considered in the following situations: antibiotic treatment with excessively broad-spectrum, inadequate CSF penetration or insufficient dosing.

The association between MMP panel use and treatment adequacy, intensive care unit admission, and in-hospital mortality was assessed using Pearson’s chi-square test of independence or Fisher’s exact test, as appropriate. Statistical analyses were performed using IBM SPSS Statistics version 23.0 (IBM Corp., Armonk, NY, USA), and a two-sided *p*-value < 0.05 was considered statistically significant.

### 2.2. Qualitative Approach

#### 2.2.1. Methodology and Population

For the qualitative approach, we used semi-structured, in-depth interviews, conducted by a clinician (O.-C.M.) between 25 February and 18 March 2025, which were further analyzed using thematic analysis. The Consolidated Criteria for Reporting Qualitative Research were used to report the methodology of the qualitative design [10]. Participants were represented by infectious disease specialists who were recruited across multiple clinical wards from our hospital through purposive sampling, in order to maximize variation in clinical seniority and case exposure, thereby aiming for obtaining accurate and rich clinical data. The infectious disease specialists interviewed were those working in both time periods.

The recruitment process took place face-to-face (16 participants) and through the phone (four participants). Consent forms were signed by all the participants before participating in the interview. All the participants were volunteers, did not receive any monetary recompensation and were not involved in the study design. The interviewer had no close personal relationship with the participants and maintained a neutral, collegial stance.

The interview was initially piloted on one person in order to ensure the fluency and adequacy of the questions, with the subsequent interview not being included in the analysis. Following the pilot interview, one question was moved earlier in the interview sequence to improve the flow of the discussion. The median duration of the interviews was 12 min. All interviews were audio recorded, transcribed verbatim by O.-C.M. and R.-M.M. and anonymized. Where quotes were used, translations were faithful to the original Romanian wording and attributed to the corresponding interview participant. Ellipses (…) were used in cited excerpts to indicate the omission of transcript segments that were not pertinent to the analytical point, ensuring conciseness while maintaining the original contextual meaning. Square brackets […] were used to introduce minor clarifications or contextual additions where necessary, without altering the substance of the participants’ statements.

#### 2.2.2. Data Collection and Analysis

Data collection was based on a predetermined interview topic guide, which was developed according to the study objectives, aiming to explore the characteristics of the meningitis cases managed, perceived strengths and weaknesses of MMP panels, the indications and clinical thresholds for MMP panels’ use, the effects of MMP panels’ results on antimicrobial therapy, as well as physicians’ broader reflections on stewardship and diagnostic strategy in such clinical scenarios.

The interview topic guide included eight questions, which are shown in Table 2. Additional questions were asked every time the interviewer considered it to be necessary, in order to ensure rich data obtainment. During the interviews, participants commonly referred to the MMP panel as “BioFire.”, because the test is marketed under the proprietary name BioFire^®^ Meningitis/Encephalitis Panel and the term “BioFire” has been widely adopted in routine clinical language to denote the test itself.

Data were analyzed using an inductive, data-driven approach of thematic analysis. We went through all the phases of thematic analysis described by Braun and Clarke [11], starting with data familiarization through repeated reading, generation of initial codes and the process of active searching for the candidate themes through grouping conceptually related codes. While several codes emerged consistently across multiple interviews and formed the core thematic structure, others appeared more sporadically and captured context-specific or divergent viewpoints. In accordance with the saliency principle, these less frequent codes were retained when they contributed to understanding physicians′ experiences and perceptions over decision-making processes in clinical practice, related to the use of MMP panels in ABM [12]. Afterwards, the themes were reviewed and refined in order to achieve both coherence and distinctiveness, while also defining and naming the themes based on their central organizing concepts. In the end, the analytic narrative was produced, while also using integrated quotes. Data saturation was obtained after 20 interviews. We stopped enrolling participants after the final interviews, since no new codes were identified and existing themes were well developed. The process of coding and the initial theme development were conducted by O.-C.M. The themes were reviewed and refined by L.-E.M. Member checking was performed by sharing the qualitative analysis with three of the interviewers, who confirmed that the interpretation accurately reflected their perspectives.

## 3. Results

### 3.1. Quantitative Approach

Group 1 included 25 (45.5%) patients whose diagnosis was established by both MMP and CSF bacterial culture, while Group 2 included 30 (54.5%) patients diagnosed exclusively through conventional bacteriologic methods. Of the total of 55 patients who met the inclusion criteria, 63.6% (*n* = 35) were male. The median age was 50 years (IQR 39–65). There were no significant differences between the two groups in terms of demographic characteristics: the proportion of male patients was 60% vs. 66.7%, (*p* = 0.6) and the median age was 48.5 years vs. 56.5 years, (*p* = 0.5).

The bacterial pathogens identified as etiological agents were *S. pneumoniae* 24 (43.6%), *L. monocytogenes* 10 (18.2%), *Staphylococcus aureus* 5 (9%), *N. meningitidis* 4 (7.2%), *H. influenzae* 3 (5.4%), *Klebsiella pneumoniae* 2 (3.6%), *S. agalactiae* 2 (3.6%) and 1 (1.8%) of *Streptococcus suis*, *Staphylococcus simulans*, *Enterococcus* spp., *Pseudomonas aeruginosa* and *Acinetobacter baumannii*. The etiological agents according to the method of identification in the two groups are shown in Table 3.

In 19 (76%) patients from Group 1, the etiological diagnosis was established by MMP and was subsequently corroborated by positive cultures in eight (30.76%) of these cases. An additional six (24%) patients in which ABM was produced by *Staphylococcus aureus*, *S. suis*, *K. pneumoniae*, *A. baumannii*, and *P. aeruginosa* were diagnosed exclusively on conventional culture methods, with the identified pathogens not being included in the MMP panel. Patients in Group 1 had a substantially (*p* < 0.05) shorter time to etiological diagnosis (median 1.9 [IQR 1.7–2.6], range 1.4–72 h) compared to Group 2 (27.3 [IQR 18–47.2] h). Clinical outcomes were similar between groups, as shown in Table 4, although the mortality was higher in Group 2.

The duration of antibiotic treatment was similar between the two groups, with a median of 14 days (IQR, 10–21) in Group 1 and 17 days (IQR, 11–22) in Group 2 (*p* = 0.07). Likewise, the length of hospitalization was comparable between groups, with a median of 22 days in both groups (IQR, 12–27 vs. 13–28.5 days, respectively) (*p* = 0.9).

Adequate empiric antibiotic therapy was more frequently prescribed in Group 1 compared with Group 2 (76% vs. 36.6%), *p* = 0.004, OR 5.46, 95% CI 1.68–17.86; regarding inadequate empiric treatment, the most frequent issue identified in both groups was the unnecessary inclusion of meropenem in the therapeutic regimens.

In Group 1, we identified 19 (76%) adequate targeted antibiotic regimens, whereas in Group 2 there were 13 (43.3%) adequate treatment regimens, *p* = 0.014, OR 4.15, 95% CI 1.29–13.33. The inadequacy of targeted antimicrobial therapy frequently involved the unjustified use of carbapenems and the administration of other combination regimens comprising broad-spectrum antimicrobial agents, including combinations with vancomycin for meningitis caused by non-resistant Gram-positive cocci.

We found that antibiotic de-escalation or optimization following the use of MMP panel or conventional microbiology results was performed in 23 patients (41.8%), with similar proportions between groups.

### 3.2. Qualitative Approach

For the qualitative part of the study, we interviewed 20 physicians, across seven different hospital wards. Participants’ age ranged from 32 to 62 years (median 37.5, IQR 35–49), while professional experience ranged from 6 to 36 years (median 12.5, IQR 10–24). The male-to-female physician ratio was 1:4, which is consistent with the male-to-female physician ratio at our institution.

Four overarching themes were identified after completing the process of analyzing and coding the transcripts, which collectively describe how clinicians perceive, interpret, and integrate MMP testing into the diagnostic and therapeutic management of ABM. The four themes are described in Table 5.

Theme 1: MMP as a Facilitator of Rapid Etiological Clarification

Clinicians consistently positioned MMP panel as a diagnostic tool that offers meaningful guidance in scenarios requiring swift decision-making. The ability to obtain an early etiological clue was described as particularly valuable during on-call hours in patients presenting with altered mental status, or when clinical history was unavailable, as illustrated below.

“From my point of view, it is very useful, especially when you are on call. When a patient presents and you have to initiate antibiotic treatment, at that moment, at 3 a.m.”

(Interview 1)

Several clinicians emphasized that the MMP panel provides orientation when conventional diagnostics are inconclusive and in diagnostically ambiguous conditions. These situations represent a major challenge from a diagnostic point of view, as well as from a therapeutic one, for every physician in real life clinical settings, and any test that may provide a piece of information meant to guide them is seen as highly beneficial.

“If the patient is immunocompromised or comes in with an unclear history, BioFire helps me orient the diagnosis early on.”

(Interview 20)

MMP panel was also regarded as especially useful in establishing the etiological diagnosis for pre-treated (“decapitated”) meningitis, due to the fact that antibiotics administered prior to lumbar puncture reduces culture sensitivity, as well as for postoperative meningoencephalitis, where establishing an etiology is often highly challenging, as it is illustrated below:

“So, it helps even in the treatment of decapitated bacterial meningitis. The patient has already started antibiotic treatment and the pathogen may no longer be detected, and then that specific fragment is identified and [it] says, yes, indeed the etiology was pneumococcal, *Listeria*, *Haemophilus*, or whatever other etiology. So, in my opinion it really helps.”

(Interview 11)

“It is useful for patients with decapitated meningitis, especially when they received antibiotics beforehand (…) I see its usefulness in postoperative meningoencephalitis… it helps narrow down what should still be considered when the result is nondetectable.”

(Interview 20)

Theme 2: Perceived Technical and Institutional Limitations

Even though the MMP system was highly appreciated among the physicians, with its speed being one of the key factors that shifted their opinion towards this direction, clinicians also emphasized several important limitations of this investigation. Most of them mentioned incomplete pathogen coverage and inconsistent sensitivity.

“Indeed, BioFire also has its limitations, such as in cases of staphylococcal meningitis, for example, because staphylococci are not included in the BioFire panel; therefore, culture and blood cultures are required, and we rely on culture-based diagnostics.”

(Interview 14)

Recurrent concerns regarded the risk of false negatives, particularly in infections caused by pathogens with lower PCR sensitivity, as well as the limited coverage of the panel for hospital-acquired meningitis, but also for relevant pathogens. Nosocomial meningitis poses a diagnostic and therapeutic challenge due to the wide spectrum of potential etiological agents, including commensal organisms and highly antibiotic-resistant pathogens. Broad empirical therapy is therefore complex and often prolonged. Consequently, because these pathogens are not included in the MMP panel, it has limited utility for etiological clarification in nosocomial meningitis, which primarily relies on conventional culture-based methods, as illustrated below.

“When it’s negative, I tend not to trust it; it may be a false negative. (…) For nosocomial pathogens, BioFire doesn’t help us; its usefulness is mainly in community-acquired cases.”

(Interview 17)

Another major concern regarded the absence of antimicrobial resistance data, which translates in fact into its inability to replace bacterial culture, which is still viewed as a cornerstone in this setting:

“It does not yet provide substantial assistance in the characterization of antibiotic resistance.”

(Interview 16)

Last but not least, institutional constraints such as limited kits and economic resources continue to represent a major issue leading to inadequate MMP use, which cannot be overcome only through clinicians’ goodwill, though necessitating major involvement from health policy makers; it is further illustrated below.

“Very often, I would like to use BioFire, but it is not available in the laboratory, as it is a relatively expensive test with limited availability.”

(Interview 1)

Theme 3: The Influence of MMP Panel’s Results on Antimicrobial De-Escalation and Stewardship

MMP panels’ results played a substantial role in clinical decision-making regarding antimicrobial therapy. Positive results were described as highly actionable, often leading to a rapid narrowing of therapy, therefore being a major factor in supporting decisions based on antibiotic stewardship principles as it is further illustrated in the following:

“If the result comes back as pneumococcus, I remove vancomycin and keep ceftriaxone.”

(Interview 17)

“Its most important impact is on stewardship—it helps us de-escalate antibiotics appropriately.”

(Interview 20)

“Because patients are often transferred from other hospitals having already received multiple antibiotics, we can no longer identify the bacterium [by culture], and it becomes very important to administer treatment directed at the specific pathogen—I do not want to prescribe meropenem to everyone. I want to target the therapy to the causative bacterium. In this situation, BioFire is extremely helpful.”

(Interview 15)

However, this was the case only for positive results, as negative ones did not lead to de-escalation, which, in clinicians’ view, makes the MMP panel an imperfect tool when it comes to implementing antibiotic stewardship practices into clinical settings, beyond guidelines:

“If the panel is completely negative, but the cerebrospinal fluid and the patient suggest that this may be bacterial meningitis, I will maintain the antibiotic treatment.”

(Interview 18)

Nevertheless, a negative result also has its utility, with participants highlighting the fact that a nondetectable result is really helpful in refocusing the differential diagnosis, which may ultimately lead, through a step-by-step process, to antibiotic optimization:

“A nondetectable BioFire helps me think about what it is not included in the panel.”

(Interview 20)

“There was this case involving *Pseudomonas*, in which I had to escalate therapy at the moment when the BioFire panel returned a nondetectable result.”

(Interview 10)

Nonetheless, the proportion of patients receiving appropriate targeted antibiotic therapy was relatively low, and many remained on broad-spectrum treatment despite an established etiology, underscoring deficiencies in antimicrobial stewardship.

Theme 4: The Integration of MMP Panel’s Result into Clinical Reasoning

Clinicians view MMP as an adjunct rather than a stand-alone diagnostic tool. Its limitations necessitate careful interpretation and continued reliance on classical diagnostics. MMP is positioned within a broader diagnostic framework that includes clinical assessment, epidemiological information, and culture-based methods.

The choice to employ MMP is influenced by more than institutional constraints, like limited kits and economic resources, as we previously mentioned. Clinicians present a contextual and selective approach to using MMP, as their decisions are also being shaped by CSF findings, clinical suspicion, and personal clinical experience. This reflects a hierarchical model of clinical reasoning, in which molecular testing supplements—but does not replace—traditional diagnostic pillars.

“The idea is that BioFire should never be considered on its own. From my point of view, it has to be integrated into the clinical and epidemiological context, as well as into the context of cultures.”

(Interview 1)

“I would not consider it a gold standard for identifying the etiology of bacterial meningitis.”

(Interview 5)

A second recurring idea is that MMP becomes particularly relevant in situations where key diagnostic inputs are missing or unobtainable. Its role is not routine but situational, emerging when clinicians cannot rely on clinical history or epidemiological data, such as in comatose patients.

“It does have utility when we are unable to determine the etiology, or when we cannot obtain any epidemiological or clinical data, for example, if the patient is comatose.”

(Interview 5)

Clinicians also frame MMP as a means of gaining time in the diagnostic process, particularly when other rapid methods fail. Importantly, this time gain is not described as an end in itself, but as a mechanism to support more rational antimicrobial use, even in the absence of resistance data.

Moreover, clinicians describe MMP as part of an explicitly staged diagnostic approach, aligned with principles of diagnostic stewardship. Its use is conditional and sequential, activated only after simpler or more traditional methods fail to provide sufficient orientation. This reflects a deliberate effort to balance technological availability with proportional use and clinical judgment.

“If I do not have an etiological diagnosis established by other rapid methods, such as latex agglutination or stained smear examination, I would want to gain time and establish the etiology using the multiplex panel, even without a resistance profile, because it can help me reduce unjustified antibiotic use. (…) I see it as useful within a staged approach, in a sort of diagnostic stewardship—if I cannot orient myself using what I have done quickly through other methods, then it might be useful.”

(Interview 16)

“If I already have a smear, let us say, and I obtain results from the smear and latex agglutination, I won’t proceed further.”

(Interview 6)

“I only consider BioFire after I see the LCR results; I don’t request it automatically.”

(Interview 18)

### 3.3. Integration of Quantitative and Qualitative Findings

The integrated analysis demonstrated strong convergence between the quantitative findings and physicians’ perceptions regarding the value of MMP panels for rapid etiological diagnosis and the optimization of antimicrobial therapy. However, a discrepancy emerged with respect to antimicrobial de-escalation, which was frequently perceived as beneficial by interviewees but was not confirmed statistically in the quantitative cohort, as shown in Table 6.

## 4. Discussion

This mixed-methods study evaluated the clinical impact of MMP panel in patients with confirmed ABM by integrating quantitative outcomes with physicians’ perspectives. To our knowledge, based on the current literature, there are no truly qualitative studies (with formal interviews, focus groups or thematic analysis) specifically exploring clinicians’ experiences or perceptions of using the MMP panel.

In terms of diagnostic stewardship, as other studies revealed, when used alongside conventional CSF analyses, MMP panels can improve both the time-to-diagnosis and the sensitivity of ABM diagnosis. In our study, the median time from lumbar puncture to diagnosis was significantly shorter when patients were diagnosed with molecular techniques (1.85 vs. 27.3 h). Similar results were reported by other papers from the literature: in a comparative study, the diagnosis using conventional methods required a mean time of approximately 13.3 (95% CI 10.7–16) h, compared to an estimated 3 h with the MMP panel [13]. In another paper, the median times to pathogen identification were 2 h using the FilmArray^®^ MMP panel and 96 h with conventional methods [14]. However, even though using MMP panel improves time-to-diagnosis, the physicians enrolled in our qualitative sub-study generally decided to resort to this method in specific scenarios, without considering it as a gold standard when it comes to diagnosing ABM. They considered MMP panel as being particularly valuable for pre-treated (“decapitated”) meningitis. In terms of diagnosis sensitivity, 11 (44%) of CSF culture-negative meningitis cases had the etiological diagnosis established using MMP panel (*S. pneumoniae*, *N. meningitidis*, and *H. influenzae* three cases each, *L. monocytogenes* and *S. agalactiae* one case each). Similarly, a study from Nigeria showed that the MMP panel detected at least one bacterial pathogen in 90 of 368 culture-negative specimens (24.5%), with *S. pneumoniae*, *N. meningitidis*, and *H. influenzae* representing the most frequently identified organisms [15]. Our quantitative analysis emphasizes the high diagnostic utility of MMP panel in cases of culture-negative meningitis, where conventional CSF cultures failed to identify a pathogen, representing 44% (11 patients) in Group 1. This is similar to other data from the literature [16,17]. However, the limitation for the positive diagnosis is for pathogens that are not included in the MMP panel, as in six (24%) patients in whom the diagnosis relied on conventional microbiology, as the bacteria were not included in the panel. A meta-analysis of MMP panel accuracy including >3000 patients demonstrated a 2% false-negative rate compared with conventional culture or specific PCR-based testing [18]. This argument was also pointed out by the physicians enrolled in the qualitative approach of our study. If a positive MMP panel was seen as highly actionable, often leading to a rapid narrowing of therapy, a negative result was considered a major limitation for this method, so as the absence of antimicrobial susceptibility profiling within the MMP panel, which is especially true for cases in which healthcare-associated meningitis were present. In clinical practice, this possibility is associated with a restriction in its utility to implement de-escalation strategies in all cases, but the negative result is still used for its high negative predictive value [19]. In terms of antimicrobial stewardship, the duration of antibiotic therapy was slightly shorter in Group 1, with a three-day reduction in median duration (14 vs. 17 days, *p* = 0.07). Data from the literature reports mixed findings in terms of the impact of the MMP panel on the total duration of antibiotic therapy. An Israeli study reported a significantly reduced duration of antimicrobial therapy in the group where multiplex PCR was used, compared to controls (eight vs. 23 patients) (9.5 ± 3.7 vs. 15.2 ± 5.0 days, *p* = 0.007) [20]. However, other two retrospective studies involving 97 and 117 patients found no difference in the total duration of antibiotic therapy [21,22].

Additionally, some data reported that narrow-spectrum antimicrobial regimens were used significantly more often in the molecular testing intervention group (78 ± 11% vs. 40 ± 9%, *p* = 0.03) [20]. Although our qualitative findings suggest that the principal advantages of using MMP panel in ABM include more rapid etiological clarification and the opportunity to optimize antimicrobial therapy when appropriate, we found that, even when the etiology was known, a relatively high proportion of patients (41.8%) remained on broad-spectrum antibiotic regimens. Notably, the proportion of patients receiving inadequate targeted antibiotic therapy was 76% in Group 1 compared to 43.3% in Group 2. While more than half of the physicians emphasized the importance of early etiological diagnosis in facilitating antimicrobial de-escalation, the treatment optimization according to the results was observed in 23 (41.8%) patients included in the study, and in 11 (44%) patients in Group 1 and 12 (40%) in Group 2. These findings draw attention to the absence of a comprehensive antimicrobial stewardship program in our setting and emphasize that more rapid availability of information does not necessarily lead to improved antimicrobial decision-making. In addition, the cost-effectiveness of molecular testing for ABM across different health systems remains poorly defined, representing an important barrier to its broader adoption in routine clinical practice.

It is unclear whether the higher proportion of appropriate therapy—both empirical and targeted—in Group 1 is attributable to the use of molecular tests, or to an improvement in prescribing antibiotics in ABM that may reflect better adherence to guidelines rather than a direct effect of molecular testing use.

In terms of clinical outcome, our quantitative research showed no significant associations between MMP panel use and length of hospitalization (22 days in both groups). MMP panel implementation was associated with modest but consistent reductions in hospital length of stay across several studies, although results are not uniform. One study showed the median hospital length of stay decreased from four (IQR 2–7) days to three (IQR 1–5) days in pre- vs. post-implementation of MMP panel (*p* = 0.03) [9]. In the same study, the implementation of the MMP panel for ABM slightly reduced the hospital length of stay compared with traditional microbiological testing methods (three vs. four days, *p* = 0.03) [9]. However, one study involving 160 adults with suspected community-onset central nervous system infections showed no significant difference in hospital length of stay between pre- and post-MMP panel periods [23]. With regard to in-hospital mortality, we found no statistically significant association between the two groups (8 vs. 20%, *p* = 0.2, OR 2.87, CI 95% 0.53–15.63), as reported by similar papers. In a study involving 97 patients with central nervous system infections, no significant mortality difference was reported between pre- and post-MMP panel groups, with overall mortality being very low [21]. No reduction in terms of in-hospital mortality was recorded in another large study involving 206 patients (2.2 vs. 2.9%, *p* = 1) [9]. It is important to recognize that outcomes are intrinsically multifactorial, and that obtaining rapid results may not necessarily influence clinical decision-making or lead to improved outcomes. It is noteworthy that, even if it did not reach statistical significance, mortality in Group 1 (including Gram-negative bacteria) (8%) was lower than in Group 2 (without Gram-negative bacillary meningitis) (20%); however, this finding may be influenced by baseline disease severity and other factors which were not captured in our retrospective analysis.

The qualitative findings provide explanatory depth and help contextualize this pattern. Despite the clear advantages for a rapid diagnosis theoretically allowing a narrow-spectrum treatment, the absence of measurable effects on length of stay, intensive care unit complications, or overall outcome suggests that improved diagnostic precision does not necessarily translate into the modification of downstream clinical endpoints. The interviewers highlight constraints such as restricted pathogen panels and the lack of resistance profiling, which may limit the test’s broader clinical impact. Moreover, physicians emphasized that disease severity at presentation, host comorbidities, and delays prior to admission play a dominant role in determining outcomes. These factors likely attenuate the potential influence of a diagnostic tool on hard clinical outcomes. The theme “Integration of MMP panel’s results into clinical reasoning” further clarifies that the MMP panel functions primarily as a decision-support instrument rather than as an independent therapeutic intervention. While it enhances clinician confidence and theoretically supports antimicrobial stewardship, it does not alter the biological trajectory of severe infection once established. This distinction between improving process measures (treatment adequacy) and modifying distal outcomes (mortality, complications) is critical for interpreting the clinical utility of rapid molecular diagnostics.

Some studies suggest that restricting MMP testing to immunocompetent adult patients with CSF abnormalities may reduce unnecessary testing and improve diagnostic yield [24,25]. A study suggested potential false-positive PCR results, as bacteria were detected in patients with normal CSF parameters and negative cultures and patients with more abnormal CSF parameters are generally more likely to have an agreement between the MMP panel’s result and bacterial culture than patients with normal CSF parameters [26]. However, our study included only patients with abnormal CSF and the proportion of culture-negative patients in Group 1 who achieved an etiological diagnosis through MMP underscores its clinical value. Notably, Group 2, which comprised patients diagnosed solely based on conventional cultures, did not include individuals with culture-negative suspected ABM. Consequently, the potential contribution of MMP panels in diagnostically challenging cases, particularly among patients with negative CSF cultures due to prior antibiotic exposure or low bacterial load, may not have been fully captured. Moreover, excluding culture- and MMP-negative cases may have reduced the observable differences between groups with regard to outcomes such as the adequacy of antimicrobial therapy, duration of hospitalization, ICU admission, or mortality. In routine clinical practice, rapid molecular testing may provide its greatest benefit precisely in cases where conventional methods fail to identify a pathogen.

Taken together, the convergent findings suggest that MMP meaningfully improves therapeutic appropriateness but has a limited impact on broader clinical outcomes in this cohort. These results underscore the importance of distinguishing between diagnostic performance, treatment optimization, and outcome modification when evaluating novel molecular technologies. Results from both the quantitative and qualitative components of the study support the complementary roles of CSF culture and MMP panels in establishing the etiological diagnosis of ABM. In the quantitative component, among patients in Group 1, 11 cases were diagnosed based solely on MMP results, whereas six cases were diagnosed exclusively by culture. Similarly, findings from the qualitative component revealed that most interviewed physicians routinely rely on both MMP panels and conventional bacteriological methods in the evaluation of meningitis cases. Together, these findings underscore the necessity of combining MMP testing with microbial culture in clinical practice.

## 5. Strengths and Limitations

A key strength of this study is the mixed-methods design, which allowed quantitative findings to be interpreted in light of clinicians’ experiential insights. However, the retrospective nature of the quantitative component, the single-center setting, and the moderate sample size may limit generalizability. Additionally, unmeasured confounding factors, including baseline disease severity, may have influenced outcome measures. Given the retrospective design of the study, a relevant outcome—time to optimal therapy—was not recorded, limiting the assessment of the impact of rapid diagnostic tests on clinical practice. Another limitation may reside in the fact that qualitative and quantitative data were not collected simultaneously, as the COVID-19 pandemic necessitated a change in the patient admission paradigm at our institute for a period of two years. Nevertheless, we attempted to mitigate this limitation by enrolling in the qualitative study physicians whose patients had been included in the quantitative assessment, in order to correlate the two types of data more accurately. Furthermore, the two study groups were drawn from different calendar periods: patients in Group 1 were treated between 2020 and 2023, whereas patients in Group 2 were admitted in our hospital in 2019. Consequently, temporal changes in clinical management, antimicrobial stewardship practices, healthcare utilization, change in local medical culture and patient case-mix may have influenced some of the observed differences between groups. Due to the limited sample size, it was not feasible to adequately evaluate the impact of the calendar year as a potential confounder through stratified analyses or multivariable adjustment for the year of admission.

To better define the appropriate integration of molecular testing into the ABM care pathway, high-quality prospective studies across diverse settings and patient populations are needed.

## 6. Conclusions

In this convergent mixed-methods study, the use of MMP panels was significantly associated with treatment optimization in patients with ABM, although the overall rate of antibiotic optimization remained low. Our qualitative findings suggested that the use of MMP enhances diagnostic certainty and may support antimicrobial de-escalation; however, its impact is constrained by technical and contextual limitations and it does not independently influence disease severity. Furthermore, our quantitative results showed that a substantial proportion of patients did not undergo de-escalation despite a known etiology, emphasizing that the more rapid availability of etiological information does not necessarily translate into improved antimicrobial decision-making.

Our findings support the incorporation of MMP into antimicrobial stewardship programs, particularly as a tool to enhance clinical decision-making and optimize antimicrobial management strategies. Future prospective studies with larger cohorts and adjustment for disease severity are needed to further clarify the downstream clinical impact of MMP in central nervous system infections.

Understanding clinicians’ experiences is essential for designing effective diagnostic pathways and for informing the future development of multiplex PCR technologies.

## Figures and Tables

**Table 1:** Adequate targeted antibiotic therapy (until final CSF culture results, if available or positive MMP panel).

Etiological Agents	Adequate Targeted Therapy
*S. pneumoniae*	Vancomycin + 3rd-generation cephalosporin
*L. monocytogenes*	Ampicillin +/− aminoglycosides
*N. meningitidis*	3rd-generation cephalosporin
*H. influenzae*	3rd-generation cephalosporin
*Streptococcus* spp.	3rd-generation cephalosporin
*Staphylococcus* spp., *Enterococcus* spp.	Vancomycin +/− aminoglycosides, Linezolid
Gram-negative bacilli (including *Pseudomonas*)	Ceftazidime or Cefepime or Meropenem

**Table 2:** Interview questions.

What is your medical specialty, and how many years of professional experience do you have?
In your clinical practice, how frequently do you treat cases of ABM?
When it comes to establishing the diagnosis of ABM, which diagnostic methods do you use in your clinical practice?
How do you select antibiotic therapy for patients with ABM?
In general, under what circumstances do you modify the antibiotic treatment?
What is your opinion regarding the investigation of the etiological diagnosis using the BioFire system? (In your practice, when do you consider using BioFire?)
In your clinical practice, how do you use the BioFire results? (Do you modify the treatment based on its results?)
I have completed the questions. Would you like to add anything further regarding the use of BioFire in the management of ABM?

**Table 3:** Bacterial pathogens identified as etiological agents in both groups, classified by the method of identification.

Etiological Agents	Group 1 *n* (%) *n* = 25		Group 2 *n* = 30 Diagnosis Based on Culture
	Diagnosis Based on MMP	Diagnosis Based on Culture	
*S. pneumoniae*	8 (42)	5 (35.7)	16 (53.3)
*L. monocytogenes*	4 (21)	3 (21)	6 (20)
*N. meningitidis*	3 (15.8)	0	1 (3.3)
*H. influenzae*	3 (15.8)	0	0
*S. agalactiae*	1 (5.2)	0	1 (3.3)
*Staphylococcus* spp.	NA	1 (7.1)	5 (16.6)
*S. suis*	NA	1(7.1)	0
*Enterococcus* spp.	NA	0	1 (3.3)
*K. pneumoniae*	NA	2 (14.2)	0
*P. aeruginosa*	NA	1 (7.1)	0
*A. baumannii*	NA	1 (7.1)	0
Total	19 (76%)	14 (56%)	30 (100)

MMP—multiplex meningitis PCR; NA—not applicable.

**Table 4:** Clinical outcome characteristics of patients with MMP panel diagnosis versus bacterial culture.

Variable	Group 1 *n* = 25	Group 2 *n* = 30	*p* Value	OR (95% CI)
Adequate empirical antibiotic treatment *n* (%)	19 (76)	11 (36.6)	0.004	5.46 (1.68–17.86)
Adequate targeted antibiotic treatment *n* (%)	19 (76)	13 (43.3)	0.014	4.15 (1.29–13.33)
Antibiotic de-escalation, *n* (%)	11 (44)	12 (40)	0.7	1.18 (0.40–3.46)
Intensive care unit admission *n* (%)	11 (44)	13 (43.3)	0.9	1.03 (0.35–3.00)
In-hospital mortality *n* (%)	2 (8)	6 (20)	0.2	2.87 (0.53–15.63)

**Table 5:** Overview of themes.

Theme Title	Theme Definition
“MMP as a facilitator of rapid etiological clarification”	This theme reflects the fact that MMP is widely perceived as an instrument that reduces diagnostic uncertainty, accelerates clinical reasoning, and supports early therapeutic direction, particularly in time-pressured situations.
“Perceived technical and institutional limitations”	This theme explores the test’s perceived limitations, thus necessitating careful interpretation and continued reliance on classical diagnostics.
“The influence of MMP panel’s results on antimicrobial de-escalation and stewardship”	This theme highlights the fact that more than half of the clinicians consider integrating MMP into antimicrobial stewardship through targeted de-escalation when results are positive, while maintaining caution toward negative results.
“The integration of MMP panel’s results into clinical reasoning”	This theme underlines that clinicians view the MMP panel as an adjunct rather than a stand-alone diagnostic tool and integrate MMP panel’s result into the diagnostic process and treatment considerations.

**Table 6:** Convergent synthesis of quantitative and qualitative findings regarding the clinical use of the MMP panel.

Theme Title	Quantitative Results	Qualitative Results	Integration
“MMP as a facilitator of rapid etiological clarification”	Patients in Group 1 had a substantially (*p* < 0.05) shorter time to etiological diagnosis compared to Group 2.	MMP reduces diagnostic uncertainty, accelerates clinical reasoning and facilitates early therapeutic decisions.	Convergence: Both data sources indicate that the MMP panel substantially accelerates etiological diagnosis.
“Perceived technical and institutional limitations”	In six patients from Group 1, the etiological diagnosis relied on conventional culture because the causative organisms were not represented in the MMP panel.	Most of the physicians mentioned the incomplete pathogen coverage; recurrent concerns were the risk of negative MMP panels and the absence of antimicrobial resistance data.	Convergence: Incomplete pathogen coverage of MMP panels
	Quantitative analysis could not evaluate factors such as availability, cost, or operational constraints.	Participants highlighted the cost and limited availability of MMP panels.	Complementarity: Qualitative findings provide contextual explanations that cannot be captured through quantitative analysis alone.
“The influence of MMP panel’s results on antimicrobial de-escalation and stewardship”	Adequate empiric antibiotic therapy and adequate targeted therapy were significantly more frequently prescribed in Group 1 compared with Group 2.	Participants reported that early identification of pathogens increased confidence in selecting appropriate antimicrobial therapy.	Convergence: Both strands suggest that MMP results contribute to more appropriate targeted therapy.
	Antibiotic de-escalation or optimization were performed in similar proportions between groups, with no statistically significant association.	Most physicians perceived the panel as useful for de-escalation and reducing unnecessary antimicrobial exposure when results are positive, while maintaining caution toward negative results.	Divergence: Clinicians perceived a stewardship benefit, although this was not demonstrated statistically in the present cohort.
“The integration of MMP panel’s results into clinical reasoning”	In Group 1, 11 patients had negative CSF cultures, whereas six patients had negative MMP results; in these cases, the etiological diagnosis was established using the alternative diagnostic method.	MMP panel is viewed as an adjunct rather than a stand-alone diagnostic tool Physicians integrate MMP panel’s result along with other tools into the diagnostic process and treatment considerations.	Convergence: MMP panels appear to be most useful when used complementary to bacterial cultures.

## Data Availability

The original contributions presented in this study are included in the article/Supplementary Material. Further inquiries can be directed to the corresponding author.

## Supplementary Materials

The following supporting information can be downloaded at https://www.mdpi.com/article/10.3390/microorganisms14061279/s1, Table S1: Empirical and targeted treatment errors, classified according to the causative pathogen; Table S2: Inadequate empirical therapy and distribution between the two study groups; Table S3: Inadequate targeted therapy, categorized by etiological agent and distributed according to study group.

## Abbreviations

The following abbreviations are used in this manuscript:

PCR	Polymerase chain reaction
MMP	Meningitis multiplex PCR
ABM	Acute bacterial meningitis
COVID-19	Coronavirus Disease of 2019
AG	Aminoglycosides
GNB	Gram-negative bacteria
NA	Not applicable
HIV	Human Immunodeficiency virus
IQR	Interquartile Range
