# Supplementary material for: The Integration of Multiplex PCR Panel in the Management of Acute Bacterial Meningitis: A Mixed-Methods Study
Source: Microorganisms. 2026 Jun 5;14(6):1279. doi: 10.3390/microorganisms14061279 (PMC13304086; doi:10.3390/microorganisms14061279)
Supplement: Supplementary file 1 [file microorganisms-14-01279-s001.zip › microorganisms-4322044-supplementary.pdf]

Supplementary Table S1. Empirical and targeted treatment errors, classified according to the causative pathogen.

Etiological agents	Inadequate empirical treatment		Reasoning	Inadequate targeted treatment		Reasoning
	Regimens	No		Regimens	No	
S. pneumoniae, N= 24	Meropenem + Vancomycin	10	Too broad spectrum	Ceftriaxone monotherapy	2	No coverage for 3rd generation cephalosporines resistant pneumococcus
	Meropenem + Linezolid	1	Too broad spectrum	Meropenem + Vancomycin	9	Too broad spectrum
	Ceftriaxone monotherapy	1	Without resistant-GPC spectrum	Meropenem + Linezolid	1	Too broad spectrum
L. monocytogenes, N=10	Ceftriaxone monotherapy	1	Without resistant-GPC spectrum	Meropenem + Ampicillin	1	Too broad spectrum
	Meropenem monotherapy	1	Broad resistant-GNB and anaerobes spectrum, no resistant-GPC spectrum	Meropenem + Linezolid	2	Too broad spectrum
	Meropenem + Vancomycin	2	Too broad spectrum	Ceftriaxone + Ampicillin	1	No benefit from adding ceftriaxone
	Meropenem + Linezolid	2				
N. meningitidis, N=4	Meropenem monotherapy	2	Broad resistant-GNB and anaerobes spectrum, no resistant-GPC Spectrum	Meropenem monotherapy	1	Too broad spectrum

H. influenzae, N=3	NA			NA		
S. agalactiae, N= 2	Meropenem + Vancomycin	1	Too broad spectrum	Meropenem + Vancomycin	1	Too broad spectrum
Staphylococcus spp., N=6	Meropenem + Vancomycin	2	Too broad spectrum	Meropenem + Vancomycin	2	Too broad spectrum
	Meropenem + Linezolid	1	Too broad spectrum	Meropenem + Linezolid	1	Too broad spectrum
Enterococcus spp., N=1	Meropenem + Linezolid	1	Too broad spectrum	Meropenem + Linezolid	1	Too broad spectrum
K. pneumoniae, N=2	-			Ceftriaxone + Amikacin	1	Not standard for possible ESBL producing *K. pneumoniae*

Supplementary Table S2. Inadequate empirical therapy and distribution between the two study groups.

Regimens	Group 1, N= 25	Group 2, N= 30	Reasoning
Meropenem + Vancomycin, N= 15	2	13	Too broad spectrum
Meropenem + Linezolid, N = 5	0	5	Too broad spectrum
Ceftriaxone monotherapy, N = 2	2	0	Without resistant- GPC spectrum
Meropenem monotherapy, N = 3	2	1	Broad resistant-GNB and anaerobes spectrum, no resistant- GPC spectrum
Total	6	19	

Supplementary Table S3. Inadequate targeted therapy, categorized by etiological agent and distributed according to study group.

Etiological agents	Regimens	Group 1, N= 25	Group 2, N= 30	Reasoning
S. pneumoniae, N= 24	Ceftriaxone monotherapy	2	0	No coverage for pneumococcus resistant to 3rd generation cephalosporines
	Meropenem + Vancomycin	0	9	Too broad spectrum

	Meropenem + Linezolid	0	1	Too broad spectrum
L. monocytogenes, N=10	Meropenem + Ampicillin	1	0	Too broad spectrum
	Meropenem + Linezolid	0	2	Too broad spectrum
	Ceftriaxone + Ampicillin	0	1	No benefit from adding ceftriaxone
N. meningitidis, N=4	Meropenem monotherapy	1	0	Too broad spectrum
S. agalactiae, N= 2	Meropenem + Vancomycin	0	1	Too broad spectrum
Staphylococcus spp., N=6	Meropenem + Vancomycin	1	1	Too broad spectrum
	Meropenem + Linezolid	0	1	Too broad spectrum
Enterococcus spp., N=1	Meropenem + Linezolid	0	1	Too broad spectrum
K. pneumoniae, N=2	Ceftriaxone + Amikacin	1	0	Not standard for possible ESBL producing *K. pneumoniae*
**Total**		6	17	
